# Genomic evidence of high gene flow and weak population structure in Siberian hazel (*Corylus heterophylla*)

**DOI:** 10.3389/fpls.2026.1844461

**Published:** 2026-05-28

**Authors:** Tae-Young Choi, Beom Kyun Park, Woong Lee, Soo-Rang Lee

**Affiliations:** 1 Department of Biology Education, College of Education, Chosun University, Gwangju, Republic of Korea; 2 Division of DMZ Forest and Biological Resources Conservation, Korea National Arboretum, Yanggu, Republic of Korea; 3 Research Institute for Dok-do and Ulleung-do Island, Kyungpook National University, Daegu, Republic of Korea

**Keywords:** Asian hazel, gene flow, genetic diversity, population structure, RAD-seq

## Abstract

Genetic diversity and connectivity strongly influence the capacity of forest tree species to persist under rapid environmental change. *Corylus heterophylla* Fisch. (Siberian hazel) is a widespread temperate shrub of ecological and economic importance in East Asia, yet genome-wide assessments of its population genetic patterns remain limited. We analyzed 4, 378 single-nucleotide polymorphisms generated from 161 individuals sampled across 20 populations in South Korea to quantify genetic diversity, population differentiation, gene flow, and demographic history. Levels of intrapopulation genetic variation were moderate and comparable to those reported for other long-lived, predominantly outcrossing tree species. Overall genetic differentiation was low (mean FST = 0.05), and isolation by distance was not detected, indicating extensive gene flow across the Korean Peninsula. Demographic reconstruction revealed two population declines during the Late Pleistocene, followed by rapid recovery and relatively stable effective population sizes toward the present. Given the limited natural dispersal capacity of the species, the observed high connectivity likely reflects long-term human-mediated movement associated with agroforestry practices. Together, these results suggest that *C. heterophylla* has maintained genetic diversity and connectivity despite historical climatic fluctuations and contemporary environmental pressures, highlighting its value as a genetic resource for conservation and breeding under ongoing climate change.

## Introductions

As dominant components of terrestrial landscapes, forest trees are experiencing unprecedented pressures from widespread habitat degradation and accelerating climatic shifts (Ledig, 1992; Nason and Hamrick, 1997; Schaberg et al., 2008). These pressures threaten the genetic diversity that underpins their long-term adaptive potential (Hamrick, 2004; Schaberg et al., 2008; Kremer et al., 2012). Trees possess distinctive life-history traits, including long generation times and extensive pollen-mediated gene flow, while seed dispersal distances vary widely and are often limited in many species (Petit and Hampe, 2006). These traits strongly influence their evolutionary responses to environmental change. Consequently, forest trees are often considered highly vulnerable to rapid environmental shifts. Long generation times can slow evolutionary responses, while the influence of high gene flow remains complex (Aitken et al., 2008; Bisbing et al., 2021). Depending on the spatial scale and selective context, gene flow may either dilute locally adapted gene complexes or facilitate adaptation by introducing beneficial alleles (Aitken et al., 2008; Bisbing et al., 2021). These combined factors can limit the pace at which populations track rapid environmental change. However, emerging evidence suggests that forest trees may be more resilient than traditionally assumed, as gene flow among populations can introduce beneficial alleles and facilitate adaptation across heterogeneous landscapes (Kremer et al., 2025). Understanding genetic diversity patterns is therefore crucial for evaluating the adaptive potential of forest tree species.

Siberian hazel (*Corylus heterophylla* Fisch., Betulaceae) is a deciduous, shade-tolerant forest shrub in temperate Asia that produces edible nuts (Molnar, 2011; Di et al., 2014). Together with *Corylus avellana* and *C. americana*, it belongs to subsection *Corylus*, characterized by leafy, overlapping involucres that enclose the nuts. Among these three species, European hazel (*C. avellana*) has received the greatest scientific attention, including extensive work on genetic diversity (Oztolan-Erol et al. 2021). This focus is due to its status as one of the world’s most commercially significant tree-nut crops, trailing only cashews, almonds, walnuts, and chestnuts (Food and Agriculture Organization of the United Nations, 2010). Although the three species share notable morphological similarities, research on *C. heterophylla* and *C. americana* remains limited (Molnar, 2011). Siberian hazel is distributed across a broad range of climates and soil types in East Asia, where it is used as a food source and as a common greening plant (Molnar, 2011). The species exhibits resistance to Eastern filbert blight and to several environmental stresses, including low temperatures and drought. These traits make it an important genetic resource for hazelnut breeding programs, especially under ongoing climate change (Molnar, 2011; Capik and Molnar, 2014). Beyond its economic value, Siberian hazel plays an important ecological role in temperate forests as an early- to mid-successional shrub whose large seed production provides an important food resource for forest fauna, supporting community stability and biodiversity (Yi and Zhang, 2008).

Siberian hazel, which often grows in close association with human activities, likely experiences distinct evolutionary and ecological processes. Although artificial selection has been less intense than in European hazel, human-mediated dispersal has expanded its distribution, creating secondary contact with otherwise allopatric congeners and facilitating hybridization under forced sympatry (Gunn et al., 2010). Unlike European hazel, which is cultivated in large agricultural systems, Siberian hazel is generally maintained in low-intensity agroforestry settings and forms diffuse populations spread across broad regions (Molnar, 2011). Such a cultivation history can shape genetic diversity patterns and influence adaptive potential (Ledig, 1992; Lefèvre, 2004). Despite its long-standing economic and ecological importance, only a small number of studies have examined genetic diversity in Siberian hazel. These studies consistently report high levels of gene flow and little evidence of genetic diversity loss (Di et al., 2014; Zhao et al., 2020), even with roughly 5, 000 years of human use (Sheng et al., 2019). However, because previous work has relied on fewer than a dozen molecular markers, the available results may not adequately reflect genome-wide patterns. A comprehensive assessment of genetic diversity using genomic-scale markers is therefore urgently needed.

The Korean Peninsula represents the southeastern edge of the mainland distribution of Siberian hazel (*Corylus heterophylla*). In this region, the species has long been harvested for edible nuts and, more recently, has been actively cultivated and improved for commercial production (Molnar, 2011). Despite this history of use and its ecological importance, population-level genomic studies of wild Korean populations remain scarce, whereas previous research has primarily addressed phylogenetic relationships across the broader range (Yang et al., 2022). Accordingly, this study addresses the following questions: (1) What is the level of genomic diversity in wild populations of Siberian hazel? (2) How is genomic variation distributed across the landscape, and to what extent are populations differentiated? and (3) Have populations experienced notable demographic changes over time? To answer these questions, we analyzed high-density genome-wide SNP markers from 161 individuals sampled across 20 populations in South Korea. We expected relatively reduced genomic diversity due to long-term human use and potential population disturbance, but also substantial gene flow resulting from anthropogenic dispersal. We also anticipated signals of demographic contraction reflecting historical land-use change and disturbance across the Korean Peninsula.

## Methods

### Study system, sample collection and DNA extraction

Siberian hazel *(Corylus heterophylla*) is a monoecious shrub that grows up to approximately 7 meters in height and spreads vigorously through abundant root suckers. The species has a chromosome number of 2n = 22 (Li and Skvortsov, 1999; Molnar, 2011). The plant produces relatively large seeds that vary in size and occur in clusters of one to seven, each enclosed within a leafy involucre. The species is native to East Asia and the Russian Far East, where it commonly grows as an understory shrub in open forests, along forest edges, on deforested hillsides, and in dry river valleys (Molnar, 2011). Like other members of *Corylus*, it is wind-pollinated, and seed dispersal is carried out primarily by small mammals, including *Apodemus*, *Clethrionomys*, and *Eutamias* (Yi and Zhang, 2008).

**Figure 1:**
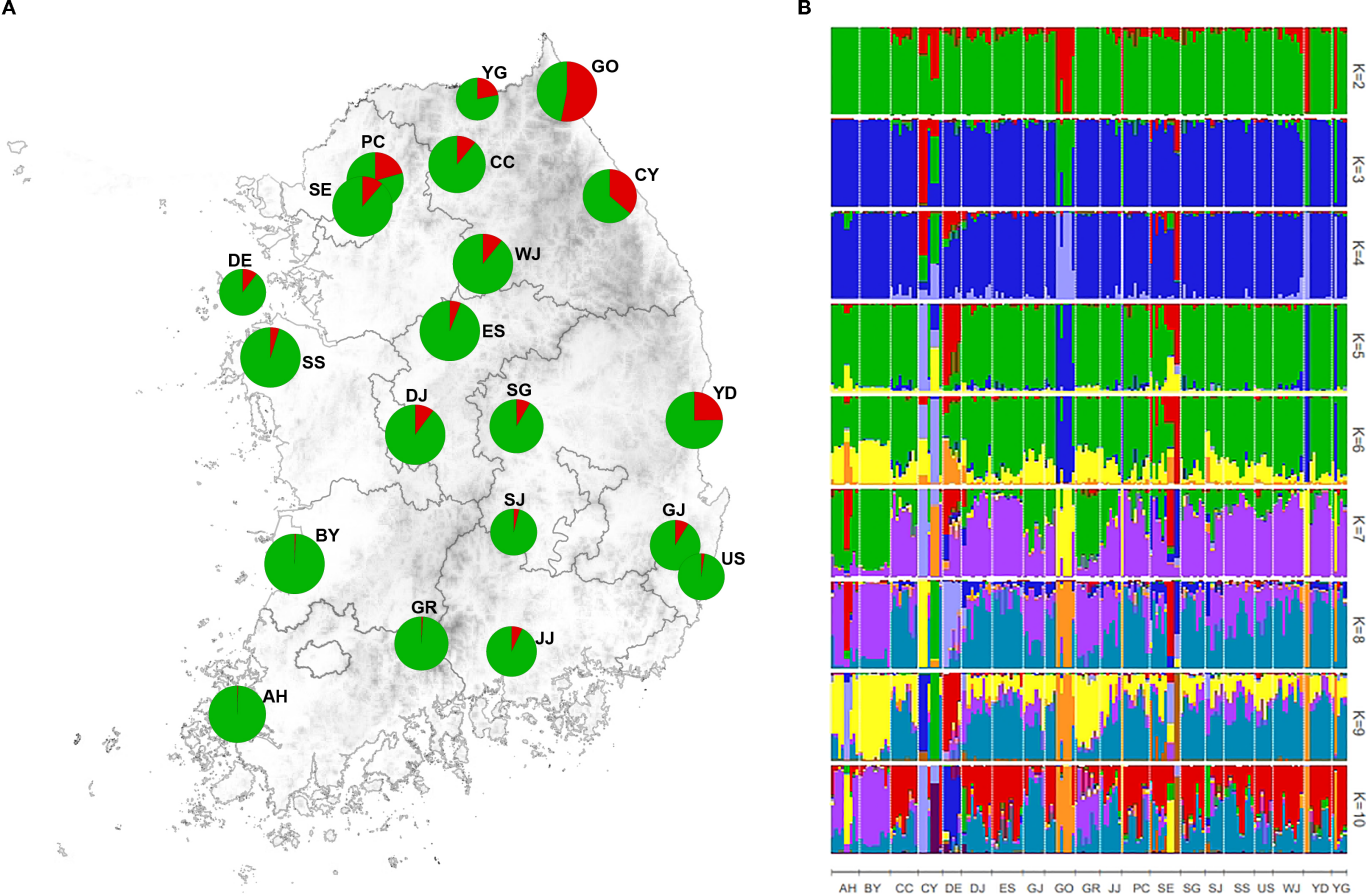
Genetic structure of 20 *Corylus heterophylla* populations from South Korea. **(a)** Pie charts for K2 (optimal K) show the proportional STRUCTURE assignment of individuals within each population. Each color represents a genetic cluster, and the proportion of each color within a pie chart corresponds to the average ancestry proportion of individuals from that population. Pie chart sizes are scaled to relative sample sizes. Grey shading represents elevation, with darker tones indicating higher elevations. **(b)** Individual assignment proportions inferred by STRUCTURE at K = 2 - 10. Populations are grouped by region and separated by dashed white vertical lines. Colors represent the proportional assignment of genotypes to each inferred genetic cluster. Population acronyms are provided in **Table 1**.

We collected samples in the summer of 2025, when Siberian hazel can be reliably distinguished from Asian beaked hazel (*C. sieboldiana*) by the degree of involucre coverage on the nuts. This distinction was essential because the geographic distributions of the two species largely overlap across South Korea. In total, 200 individuals were sampled from 20 populations distributed across South Korea (**Table 1**, **Figure 1a**). Given the population-level sampling design of this genetic study, one representative individual per population was selected for voucher specimen preparation. Detailed voucher information, including the full name of the herbarium and accession numbers, is provided in **Supplementary Table 1**, and specimen identification was carried out jointly by the authors in the field. All voucher specimens were deposited in a public herbarium (CHO). Sampling sites were separated by a minimum distance of 30 km to reduce the likelihood of sampling individuals from the same genetic group, given the species’ wind mediated pollen and animal assisted seed dispersals. This sampling design was planned to capture regional-scale genetic structure rather than fine-scale spatial patterns. Individual plants within each population were collected at least 50 m apart to ensure avoiding genetically close relatives. All fresh leaf tissues were immediately dried in silica gel for DNA extraction.

**Table 1:** Summary of genetic diversity indices for *Corylus heterophylla* populations estimated from 4, 378 SNPs across sampling sites.

Location	Population acronym	No. of samples	GPS coordinates		Ho	He	Ar_rare	Fis [CI]
			Longitude	Latitude				
Jeollanam-do, Sinan-gun, Aphae-eup	AH	7	126.260319	34.847952	0.23 [0.16]	0.26 [0.14]	1.42 [0.35]	0.04[0.01, 0.06]
Jeonbuk-do, Buan-gun, Sangseo-myeon	BY	10	126.634285	35.681072	0.23 [0.16]	0.26 [0.14]	1.45 [0.34]	0.04[0.02, 0.06]
Gangwon-do, Chuncheon-si	CC	9	127.739822	37.888744	0.22 [0.15]	0.25 [0.13]	1.47 [0.32]	0.04[0.02, 0.06]
Gangwon-do, Pyeongchang-gun, Daegwallyeong-myeon	CY	8	128.799012	37.706824	0.31 [0.21]	0.32 [0.14]	1.38 [0.40]	-0.03[-0.06, 0]
Incheon, Ongjin-gun, Jawol-myeon	DE	6	126.259954	37.176986	0.33 [0.21]	0.35 [0.13]	1.42 [0.42]	-0.02[-0.05, 0.01]
Daejeon, Daedeok-gu, Jangdong-ro	DJ	10	127.44975	36.39736	0.22 [0.15]	0.25 [0.13]	1.48 [0.32]	0.05[0.03, 0.07]
Chungcheongbuk-do, Eumseong-gun, Eumseong-eup	ES	9	127.687065	36.970177	0.21 [0.14]	0.24 [0.13]	1.47 [0.31]	0.07[0.05, 0.09]
Gyeongsangbuk-do, Gyeongju-si, Naenam-myeon	GJ	7	129.209427	35.775105	0.25 [0.16]	0.29 [0.13]	1.45 [0.37]	0.04[0.02, 0.06]
Gangwon-do, Goseong-gun, Toseong-myeon	GO	10	128.509169	38.286859	0.20 [0.14]	0.26 [0.14]	1.46 [0.33]	0.1[0.08, 0.12]
Jeollanam-do, Gurye-gun, Masan-myeon	GR	7	127.492332	35.244743	0.24 [0.16]	0.27 [0.13]	1.45 [0.35]	0.05[0.03, 0.08]
Gyeongsangnam-do, Jinju-si	JJ	7	128.097778	35.199463	0.24 [0.15]	0.28 [0.13]	1.47 [0.36]	0.08[0.05, 0.1]
Gyeonggi-do, Pocheon-si, Soheul-eup	PC	9	127.170016	37.798361	0.21 [0.14]	0.25 [0.13]	1.48 [0.32]	0.08[0.06, 0.11]
Seoul, Nowon-gu, Junggye-dong	SE	10	127.082924	37.656017	0.23 [0.16]	0.27 [0.14]	1.44 [0.35]	0.05[0.03, 0.08]
Gyeongsangbuk-do, Sangju-si, Yeonwon-dong	SG	8	128.141228	36.442331	0.23 [0.15]	0.26 [0.13]	1.48 [0.34]	0.06[0.04, 0.08]
Gyeongsangbuk-do, Seongju-gun, Gacheon-myeon	SJ	6	128.117355	35.858089	0.28 [0.19]	0.32 [0.13]	1.42 [0.40]	0.05[0.02, 0.08]
Chungcheongnam-do, Seosan-si, Seongyeon-myeon	SS	10	126.456474	36.819359	0.22 [0.15]	0.25 [0.13]	1.47 [0.33]	0.04[0.02, 0.06]
Ulsan, Buk-gu	US	6	129.381626	35.598517	0.26 [0.17]	0.30 [0.13]	1.45 [0.38]	0.05[0.02, 0.07]
Gangwon-do, Wonju-si, Sicheong-ro	WJ	8	127.916809	37.33923	0.21 [0.15]	0.24 [0.13]	1.45 [0.32]	0.06[0.04, 0.08]
Gyeongsangbuk-do, Yeongdeok-gun, Yeongdeok-eup	YD	9	129.354492	36.462378	0.21 [0.16]	0.26 [0.13]	1.42 [0.35]	0.1[0.07, 0.12]
Gangwon-do, Yanggu-gun, Bangsan-myeon	YG	5	127.882461	38.249017	0.28 [0.17]	0.33 [0.13]	1.47 [0.41]	0.06[0.03, 0.08]

Ho, He, and Ar_rare denote observed heterozygosity, expected heterozygosity, and allelic richness standardized for sample size, respectively. Values in brackets indicate standard errors, and CI refers to confidence intervals.

Genomic DNA was extracted using a modified CTAB protocol (Doyle and Doyle, 1987), with further adjustments implemented due to the high phenolic content of *Corylus* tissues, which is known to interfere with DNA isolation (Riethmüller et al., 2013). Silica-dried leaf tissues were homogenized using a Lysera system (Biotage, Sweden) with silica beads, followed by an additional sorbitol wash step to remove soluble contaminants (Schenk et al., 2023). The CTAB extraction buffer was supplemented with 1%(w/v) PVP40 and 0.5%(v/v) β-mercaptoethanol to bind and neutralize phenolic compounds. Samples were incubated for 1 h at 65 °C with RNaseA prior to organic extraction and DNA precipitation.

### RAD-seq library preparation and raw data processing

Genomic DNA was genotyped using the 2RAD method (Bayona-Vásquez et al., 2019), a modification of the widely adopted ddRAD approach (Peterson et al., 2012). Library preparation followed Bayona-Vásquez et al. (2019) with minor adjustments. Genomic DNA was first digested with the frequent cutter, EcoRI-HF (Thermo Fisher Scientific), and a rare cutter, XbaI (Thermo Fisher Scientific). After digestion, adapter ligation, pooling, and cleanup were completed. Fragment sizes of 450–500 bp were selected using a Pippin Prep system (Sage Science, Beverly, MA). PCR amplification was then performed, followed by purification of the amplicons. All cleanup steps used a 1:1.8 ratio of AMPure XP beads and a 70% ethanol wash. Final library quality was evaluated with an Agilent 2100 Bioanalyzer (Agilent Technologies, Santa Clara, CA, USA). Libraries were sequenced on an Illumina NovaSeq platform at Macrogen Inc. (Seoul, Korea) using 2 × 150 bp paired-end reads.

Raw reads were demultiplexed and trimmed to 141 bp using the process_radtags module in Stacks v2.41 (Rochette et al., 2019). Low-quality reads were filtered using a Phred threshold of 10 within a 0.15 sliding window. High-quality reads were mapped to the *C. heterophylla* reference genome (GCA_016403345.1) with Bowtie v. 2.2.3 (Langmead and Salzberg, 2012) using the --very-sensitive option, and only alignments with MAPQ ≥ 30 were retained using Samtools (Li et al., 2009). RAD loci were identified and assembled using the ref_map.pl pipeline in Stacks. SNP calling was conducted with the populations module, requiring presence in at least 10 populations (-p 10) and in at least 70% of individuals (-r 0.7). To reduce linkage, only the first SNP per RAD locus was retained. Quality control filtering included removing SNPs showing significant deviation from Hardy–Weinberg equilibrium (P < 1 × 10^-6^) using Plink v1.9 (Purcell et al., 2007), and excluding genotypes with high missingness as well as SNPs with a minor allele frequency (MAF) ≤ 0.1 using TASSEL v5.0 (Glaubitz et al., 2014).

### Genetic diversity and population structure

We used Arlequin v3.5 (Excoffier and Lischer, 2010) to calculate observed (Ho) and expected (He) heterozygosities from the final dataset of 4, 378 SNPs. To account for unequal sample sizes, allelic richness (Ar) was estimated using rarefaction in HP-Rare (Kalinowski, 2004; Kalinowski, 2005). The inbreeding coefficient (F_IS_) was calculated with the R packages adegenet v2.1.11 and vcfR v1.15.0 (Jombart and Ahmed, 2011; Knaus and Grünwald, 2017). We identified locally common alleles following the criteria of van Zonneveld et al (Van Zonneveld et al., 2012). The number of locally common alleles per locus was calculated in GENALEX v6.5.1 (Peakall and Smouse, 2012). The Pairwise FST values among the 20 populations were also estimated in Arlequin, with statistical significance assessed using 1, 000 permutations. We visualized genomic differentiation for two representative population pairs: one with the highest genome-wide mean FST and another with a mean FST closest to the overall average. Windowed FST was calculated using the Weir and Cockerham (1984) estimator in VCFtools (Danecek et al., 2011) with 2 Mb sliding windows and a 0.5 Mb step size across the reference genome. The resulting values were plotted along cumulative chromosomal coordinates as Manhattan-style plots using ggplot2 (Wickham, 2016). To test whether population differentiation was significantly correlated with geographic distance, we conducted a Mantel test using log-transformed Euclidean distances as predictors and linearized FST [FST = FST/(1 − FST)] in GENALEX (Rousset, 1997).

Population genetic structure was examined using the 4, 378 unlinked SNPs through two complementary approaches. First, principal component analysis (PCA) was performed in R using the glPca function in the adegenet package (Jombart, 2008). The first three principal component pairs (PC1–PC2, PC1–PC3, and PC2–PC3) were plotted to assess patterns of genetic differentiation among individuals. Second, the number of genetic clusters (K) was inferred using STRUCTURE v. 2.3.4 (Pritchard et al., 2000) as implemented in ipyrad. Analyses were performed under an admixture model with 300, 000 Markov Chain Monte Carlo (MCMC) iterations following a burn-in period of 60, 000 steps. We evaluated K values from 1 to 10, with 10 independent runs per K to assess consistency among replicates. The optimal K was determined using the ΔK method (Evanno et al., 2005) implemented in STRUCTURE HARVESTER v. 0.6.93 (Earl and VonHoldt, 2012). Replicate runs for the selected K were aligned and summarized using CLUMPP v. 1.1.2 (Jakobsson and Rosenberg, 2007), and the resulting ancestry coefficients were visualized with the R package pophelper v. 2.3.1 (Francis, 2017). In addition to assignment-based approaches, we explored patterns of gene flow among populations using TreeMix v1.12 (Pickrell and Pritchard, 2012). The dataset, consisting of 4, 378 SNPs, was converted into TreeMix input format using the populations module in Stacks. Because no suitable outgroup was available, trees were inferred without rooting. To assess potential migration, we performed 500 bootstrap replicates with a SNP block size of 100 (using the -K option) and evaluated models allowing between zero and five migration edges (m = 0–5). The optimal number of migration events was determined using the R package OptM (Fitak, 2021).

In addition, we used Stairway Plot v2.0 (Liu and Fu, 2020) to infer recent changes in effective population size (Ne) for the Korean population of *C. heterophylla*. Individuals showing evidence of admixture were excluded to better satisfy the assumptions of site frequency spectrum–based demographic inference, which assume a single, panmictic population and can be biased by population structure or admixture (Liu and Fu, 2020). We only included genotypes that are assigned to the green genetic cluster on the STRUCTURE result (**Figure 1**). For input preparation, folded site frequency spectrum (SFS) was generated with easySFS (https://github.com/isaacovercast/easySFS (Gutenkunst et al., 2009);) and the maximum number of segregating sites per population was selected from projection values. We performed 200 bootstrap replicates and applied a mutation rate of 3.75 × 10^-8^ substitutions per site per generation, assuming a 4-year generation time (Molnar, 2011; Yang et al., 2023).

## Results

Each of the 200 samples yielded an average of 14 million raw sequence reads that passed Illumina quality filters. The mean GC content and coverage were 40.5% and 186 x respectively. Following demultiplexing and trimming, 39 samples were excluded due to missing or low-quality RAD cut sites. After a series of pruning steps, we identified 4, 378 high-quality SNPs for the 161 pooled samples. Consistent with the filtering strategy of retaining one SNP per RAD locus, these SNPs were broadly distributed across the genome (**Supplementary Figure 1**).

**Figure 2:**
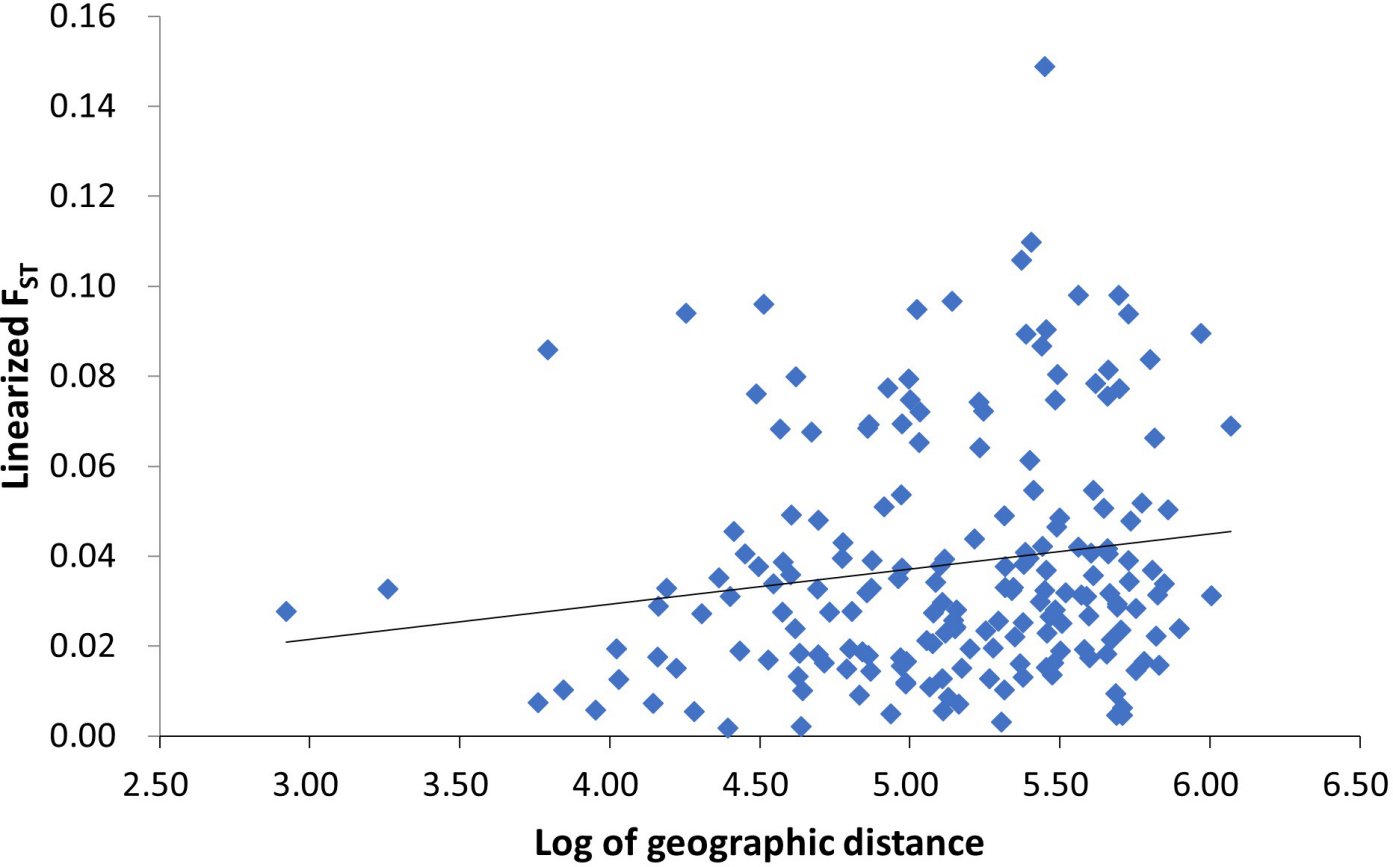
Mantel test results showing the relationship between log-transformed Euclidean geographic distance (km) and Slatkin’s linearized FST [FST/(1 − FST)] among 20 populations of *Corylus heterophylla* from South Korea (r = 0.16, P > 0.05).

Overall, genetic diversity varied among populations, with mean values of Ho = 0.24, He = 0.28, and Ar = 1.5, although these differences were minor (**Table 1**). The ES and WJ populations exhibited the lowest He, whereas YG showed the highest He (**Table 1**). The number of alleles standardized for population size ranged from 1.38 in CY to 1.48 in DJ, PC, and SG (**Table 1**). No substantial inbreeding was detected, as inbreeding coefficients were below 0.1 in all populations (**Table 1**). Genetic differentiation was low, with a mean FST of 0.05 and pairwise values ranging from 0.001 (e.g., DD-PC and PC-YG) to 0.16 (CY-DE, **Table 2**). Most pairwise FST values were below 0.05, indicating weak differentiation among the majority of population pairs. Increased differentiation was primarily associated with population pairs involving the CY and DE, which showed consistently higher FST values relative to those among most other population pairs, with a mean FST of 0.10 (**Table 2**; **Supplementary Figure S2**). In contrast, many population pairs exhibited near-zero FST values, suggesting minimal genetic differentiation. This pattern partially corresponds with the clustering inferred from the STRUCTURE analysis (**Figure 1**). To examine the genomic distribution of differentiation, we generated Manhattan plots of per-locus FST for two representative comparisons: CY vs DE (high differentiation) and US vs SE (typical differentiation). In both cases, FST values were broadly distributed across the genome rather than concentrated in pronounced peaks, with most loci showing moderate differentiation and only a small number of outliers (**Supplementary Figure 3**). The Mantel test indicated a weak correlation between genetic differentiation and geographic distance (r = 0.16), and this relationship was not statistically significant (P > 0.05; **Figure 2**). Taken together, the predominance of low pairwise FST values and the absence of IBD indicate weak population differentiation.

**Table 2:** Mean pairwise FST values estimated from 4, 378 SNPs among 20 populations of *Corylus heterophylla*.

	AH	BY	CC	CY	DE	DJ	ES	GJ	GO	GR	JJ	PC	SE	SG	SJ	SS	US	WJ	YD	YG
AH	0.000																			
BY	0.044	0.000																		
CC	0.035	0.025	0.000																	
CY	0.110	0.105	0.078	0.000																
DE	0.121	0.105	0.088	0.159	0.000															
DJ	0.045	0.038	*0.012*	0.078	0.085	0.000														
ES	0.036	0.029	*0.006*	0.077	0.083	0.020	0.000													
GJ	0.055	0.043	0.026	0.100	0.103	0.032	0.028	0.000												
GO	0.095	0.081	0.043	0.117	0.114	0.055	0.051	0.066	0.000											
GR	0.032	0.024	0.014	0.093	0.095	*0.025*	0.019	0.035	0.075											
JJ	0.038	0.029	0.013	0.090	0.093	*0.021*	*0.008*	0.029	0.057	0.067	0.000									
PC	0.040	0.031	*0.007*	0.081	0.078	0.015	0.013	0.032	0.032	0.034	*0.015*	0.000								
SE	0.064	0.061	0.038	0.111	0.111	0.044	0.039	0.062	0.073	0.084	0.044	0.038	0.000							
SG	0.036	0.035	0.011	0.080	0.078	*0.011*	0.012	0.033	0.046	0.060	0.014	*0.010*	0.041	0.000						
SJ	0.069	0.056	0.040	0.111	0.117	0.044	0.040	0.060	0.078	0.102	0.038	0.041	0.073	0.041	0.000					
SS	0.046	0.039	0.018	0.092	0.096	0.031	0.023	0.039	0.060	0.068	*0.022*	0.024	0.053	0.028	0.048	0.000				
US	0.042	0.034	0.014	0.091	0.096	0.027	0.017	0.045	0.073	0.088	*0.019*	0.021	0.048	0.025	0.055	0.032	0.000			
WJ	0.036	0.036	*0.007*	0.078	0.085	*0.012*	*0.008*	0.032	0.044	0.076	0.011	*0.002*	0.040	0.010	0.045	0.019	0.021	0.000		
YD	0.056	0.047	*0.021*	0.099	0.100	0.029	0.021	0.047	0.050	0.076	0.031	*0.021*	0.058	*0.025*	0.058	0.035	0.040	0.059	0.000	
YG	0.047	0.038	*0.009*	0.092	0.087	*0.022*	0.017	0.034	*0.033*	0.052	0.025	*0.001*	0.044	*0.011*	*0.042*	0.031	0.026	*0.033*	*0.027*	0.000

Population acronyms are defined in **Table 1**. Values shown in red are not statistically significant (P > 0.05), based on 1, 000 permutations. P values of each estimate is provided in **Supplementary Table 2**.

**Figure 3:**
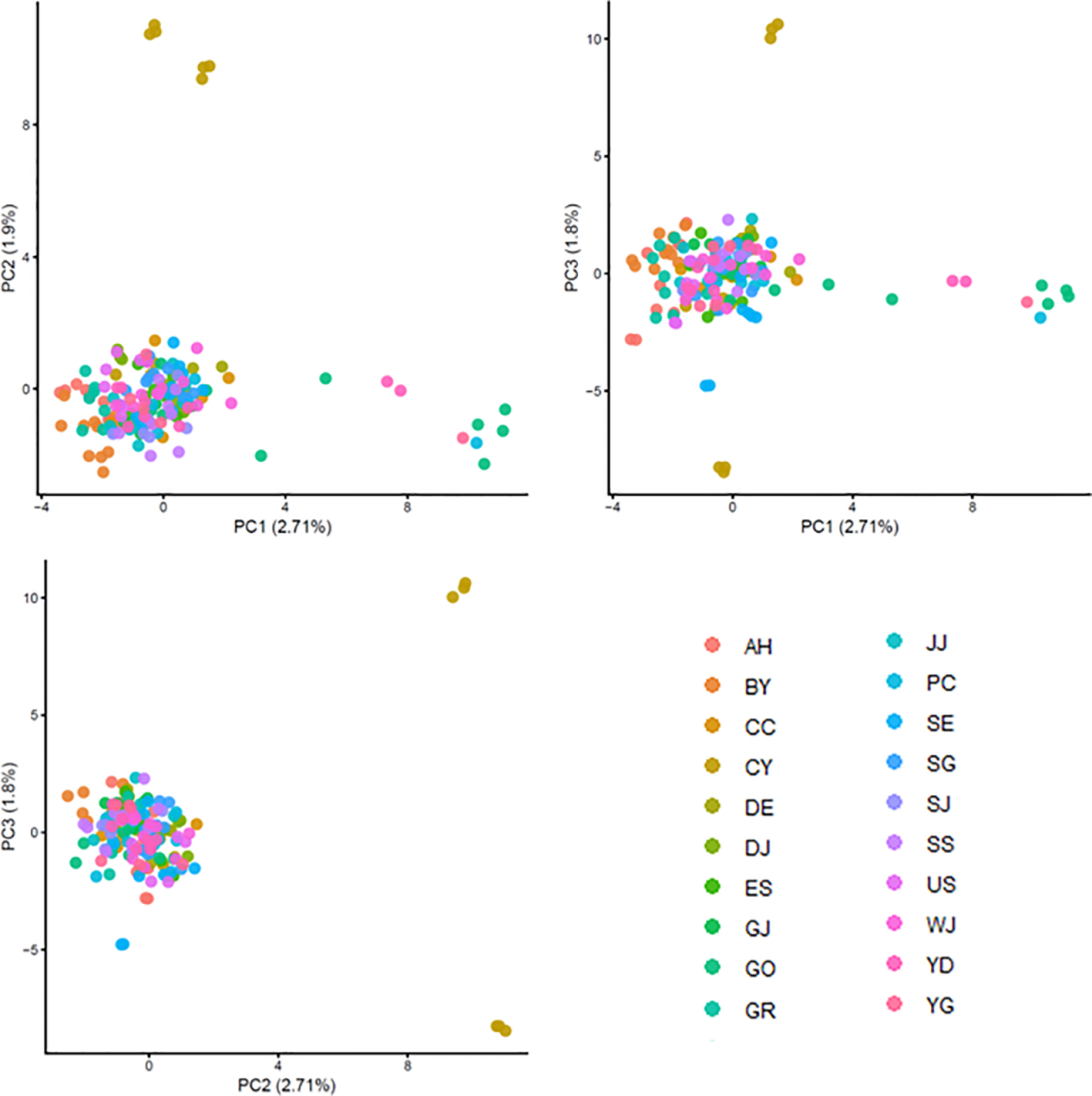
Result of principal coordinate analysis of 20 *Corylus heterophylla* populations.

In the principal component analysis (PCA), the first three principal components explained 2.7%, 1.9%, and 1.8% of the total genetic variation, respectively (**Figure 3**). This low variance explained is consistent with weak population structure. Although the variance explained was low, the overall clustering pattern broadly matched the STRUCTURE results (**Figures 1**, **3**). Most genotypes formed a single diffuse cluster, while individuals from CY were more divergent and tended to separate from the main group. In addition, a subset of genotypes from GO, PC, YD, and YG showed partial separation along the PC1 axis. These populations also displayed relatively higher proportions of the minor genetic cluster in the STRUCTURE analysis at K = 2 (**Figures 1**, **3**; **Supplementary Figure S2**). However, given that the first three principal components together accounted for less than 7% of the total variance, the PCA patterns should be interpreted cautiously.

In the STRUCTURE analysis, the optimal number of genetic clusters was 2, as inferred using the delta K method (**Supplementary Figure 2**). We selected K = 2 as the best fitting model for presentation because it best captured biologically meaningful patterns in *C. heterophylla*. Results for alternative K values are provided in **Figure 1b**. At K = 2, allele assignment patterns showed no clear association with geographic relationships within Korea, which is consistent with the Mantel test results (**Figures 1**, **2**). The bar plot revealed a very limited number of admixed individuals, indicating a low likelihood of recent admixture events (**Figure 1b**). At the best K, individuals were primarily assigned to a dominant genetic cluster with a secondary cluster occurring at lower frequencies across populations (**Figure 1**). Overall, we found two genetic clusters represented by green and red. The two genetic clusters inferred at K = 2 (hereafter referred to as Cluster 1 and Cluster 2) correspond to the green and red components in the STRUCTURE plot (**Figure 1**), representing individual ancestry proportions (Q values provided in **Supplementary Information 2**). Most individuals were predominantly assigned to Cluster 1 (Q > 0.8), while Cluster 2 was present at lower frequencies across populations, with relatively higher representation in northeastern populations (**Figure 1**; **Supplementary Information 2**). TreeMix analysis did not reveal clear or consistent migration edges, and the inferred population relationships were poorly resolved (**Supplementary Figure 4**).

**Figure 4:**
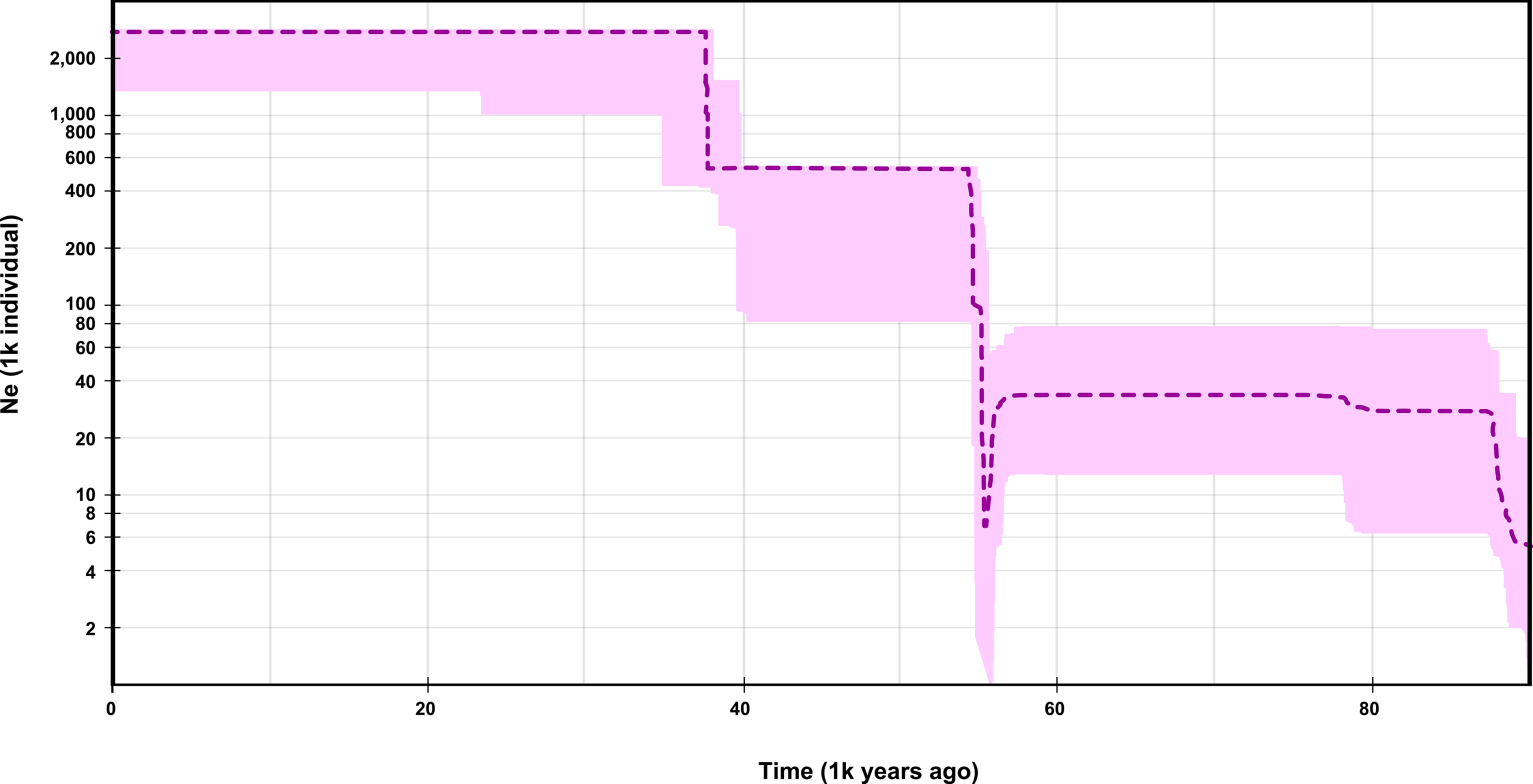
Historical changes in effective population size (Ne) of the South Korean *Corylus heterophylla* population inferred from Stairway Plot analysis. Shaded areas indicate the 95% confidence interval.

The effective population size of the current Korean population, estimated using Stairway Plot analysis, was slightly over 2 million individuals (**Figure 4**). The inferred demographic history of Korean *C. heterophylla* revealed multiple changes in effective population size through time. An initial population decline was detected around 80 ka, followed by a period of moderate recovery (**Figure 4**). Around 40 ka, effective population size declined again, indicating a second population bottleneck (**Figure 4**). Subsequently, Ne increased rapidly and remained relatively stable toward the present.

## Discussion

Despite its ecological and economic importance in temperate forest ecosystems, Siberian hazel (*Corylus heterophylla*) has received relatively little attention in population genomic studies (Molnar, 2011). In the context of widespread anthropogenic disturbance affecting forest systems, this species has likely experienced multiple environmental stressors with potential consequences for genetic diversity and adaptive capacity. Using a population genomics framework across heterogeneous landscapes, we detected high levels of gene flow, with weak population structure approaching panmixia among Korean populations suggesting that human-mediated dispersal may have contributed to contemporary genetic structure. Notably, genetic diversity has remained comparatively well preserved despite evidence for two severe demographic bottlenecks during the Late Pleistocene. Collectively, these results suggest that *C. heterophylla* has maintained substantial evolutionary resilience under both historical climatic fluctuations and ongoing environmental pressures, underscoring its value as a genetic resource for conservation and breeding efforts in the face of rapid climate change.

Genetic diversity is a key component of the adaptive potential and long-term persistence of forest tree species, and its erosion is widely regarded as a major threat to forest ecosystems (Schaberg et al., 2008). However, population-level assessments of genetic diversity in *Corylus heterophylla* remain limited, and direct comparisons among previous studies are hindered by the use of different marker systems and relatively small numbers of loci (Di et al., 2014; Zhao et al., 2020; Yang et al., 2022). Although Yang et al. (2023) reported genetic diversity estimates similar to those observed here, their analysis was based on a limited sample size of approximately 30 individuals. Using a substantially larger dataset of more than 160 individuals, our genomic analysis indicates that Korean *C. heterophylla* populations maintain moderate to high levels of intrapopulation genetic variation (mean He = 0.28). These values are comparable to those reported for closely related species, including *C. avellana* [He = 0.28 (Armstrong et al., 2024)], *C. cornuta* [He = 0.20 (Jiang et al. 2017)], and *C. americana* [He = 0.28 (Revord et al., 2020)], as well as for other long-lived, predominantly outcrossing tree species such as Scots pine (*Pinus sylvestris*; He = 0.26 (Bruxaux et al., 2024);). Despite evidence for at least two periods of reduced effective population size during the late Pleistocene (ca. 80–40 ka; **Figure 4**), contemporary populations exhibit levels of genetic diversity comparable to those of related taxa. This pattern indicates that genetic variation has persisted despite historical demographic fluctuations, although the relative contributions of life-history traits, demographic history, and geographic distribution cannot be fully disentangled with the present data.

Alternatively, the relatively high genetic variation observed in *C. heterophylla* may be partly attributable to long-term human-mediated influences. Anthropogenic management has been shown to shape the genetic composition of forest tree populations, as illustrated by elevated levels of genetic diversity in wild trees maintained within traditional agroforestry systems, such as rustic coffee plantations (Bandeira et al., 2005). Although Siberian hazel has not experienced the intensive cultivation characteristic of European hazel, it has been incorporated into low-intensity agroforestry systems for centuries (Molnar, 2011). Recent studies have further demonstrated that traditionally managed plant populations can serve as reservoirs of genetic variation in crop and semi-domesticated species (Miller and Schaal, 2006; Jarvis et al., 2008; Gunn et al., 2010; Rivas et al., 2023). In Korea, the cultivation of Siberian hazel has been promoted by government programs as a supplemental income source for local farmers and as a greening species in urban parks, resulting in managed populations distributed across the country (Kim and Yoon, 1983). Such scattered, managed populations may have contributed to the maintenance of genetic diversity in wild populations by enhancing population persistence and connectivity, although the magnitude of this effect in *C. heterophylla* remains to be evaluated.

Despite its broad geographic distribution across diverse environmental gradients (Molnar, 2011), which would typically be expected to promote genetic differentiation among populations [e.g (Duffy, 2024)], *Corylus heterophylla* exhibits unexpectedly high levels of gene flow across the Korean Peninsula. This is reflected in near-zero FST values (**Table 2**) and the absence of isolation by distance. Such patterns are difficult to reconcile with the species’ natural dispersal biology. Seed dispersal in Siberian hazel is largely restricted to short-distance movements by small rodents (Yi and Zhang, 2008), and although wind-mediated pollen dispersal can occur over greater distances, most effective pollen exchange in wind-pollinated trees is limited to several hundred meters to a few kilometers, with long-distance events being rare (Bittencourt and Sebbenn, 2007; Pluess et al., 2009; Kremer et al., 2012). Consequently, natural dispersal alone is unlikely to fully account for the near-panmictic pattern observed. Because our sampling design targeted populations separated by at least 30 km, the results primarily reflect regional-scale genetic structure, and finer-scale spatial differentiation cannot be excluded. Consistent with this interpretation, per-locus FST values were broadly distributed across the genome with few pronounced outliers (**Supplementary Figure 3**), indicating diffuse differentiation rather than strong localized genomic divergence. Comparable levels of gene flow have been reported in other tree crops, including walnut [mean FST = 0.09 (Gunn et al., 2010)] and chestnut [*Castanea dentata*, mean FST = 0.07 (Sandercock et al., 2022), *Castanea sativa*, mean FST = 0.09 (Bouffartigue et al., 2020)], where human-mediated movement has enhanced connectivity. Together, these results suggest that long-standing agroforestry practices on the Korean Peninsula may have contributed to reduced isolation by distance, contributing to the near-zero FST values observed in Korean populations of Siberian hazel.

Spatial genetic structure in Korean Siberian hazel was weak in the STRUCTURE analysis, aligned with the high levels of gene flow inferred among regional populations and patterns reported in other *Corylus* species (Revord et al., 2020; Zhao et al., 2020; Wang et al., 2023). Although most populations were dominated by a shared genetic background, a secondary genetic component was detected at low to moderate frequencies across Korea, with relatively higher representation in northeastern populations. This pattern may reflect a localized reservoir of genetic variation. Northern and eastern Korea are characterized by complex mountainous terrain and pronounced environmental heterogeneity (Kwon et al., 2016; IAEA, 2023), which may have facilitated the persistence of regionally differentiated lineages or limited local gene flow. TreeMix analysis likewise did not identify clear migration edges and produced a poorly resolved topology, as expected under conditions of weak population structure where allele frequency differences among populations are minimal and allele frequency-based methods have limited power to infer directional gene flow. These results have important implications for the conservation and utilization of *C. heterophylla*. High genetic diversity and weak population structure indicate broadly distributed genetic resources across the Korean Peninsula, while regionally enriched components emphasize the need to conserve populations across heterogeneous environments. Together, these patterns highlight the species’ value as a genetic resource for conservation and hazelnut improvement under ongoing environmental change.

## Data Availability

The datasets presented in this study can be found in online repositories. The names of the repository/repositories and accession number(s) can be found in the article/**Supplementary Material**.
