# Supplementary material for: Genomic evidence of high gene flow and weak population structure in Siberian hazel (Corylus heterophylla)
Source: Front Plant Sci. 2026 May 28;17:1844461. doi: 10.3389/fpls.2026.1844461 (PMC13253746; doi:10.3389/fpls.2026.1844461)
Supplement: Supplementary file 1 [file Table1.docx]

Supplementary Information

Genomic evidence of high gene flow and weak population structure in Siberian hazel (*Corylus heterophylla*)

Tae-Young Choi^1^, Beom Kyun Park^2^, Woong Lee^3^ and Soo-Rang Lee^1*^

^------------------------------------------------------------------------------------------------^

^1^Department of Biology Education, College of Education, Chosun University, Gwangju 61452, South Korea

^2^Division of DMZ Forest and Biological Resources Conservation, Korea National Arboretum, Yanggu 24564, Republic of Korea

^3^Research Institute for Dok-do and Ulleung-do Island, Kyungpook National University, Daegu 41566, South Korea

Table S1. Voucher specimen information for representative individuals sampled from each population in this study.

| Location | Population acronym | Voucher number | Deposition |
| --- | --- | --- | --- |
| Jeollanam-do, Sinan-gun, Aphae-eup | AH | CHAH2518 | Chosun University Herbarium (CHO) |
| Jeonbuk-do, Buan-gun, Sangseo-myeon | BY | CHBY2512 | Chosun University Herbarium (CHO) |
| Gangwon-do, Chuncheon-si | CC | CHCC2518 | Chosun University Herbarium (CHO) |
| Gangwon-do, Pyeongchang-gun, Daegwallyeong-myeon | CY | CHCY2508 | Chosun University Herbarium (CHO) |
| Incheon, Ongjin-gun, Jawol-myeon | DE | CHDE2507 | Chosun University Herbarium (CHO) |
| Daejeon, Daedeok-gu, Jangdong-ro | DJ | CHDJ2518 | Chosun University Herbarium (CHO) |
| Chungcheongbuk-do, Eumseong-gun, Eumseong-eup | ES | CHES2518 | Chosun University Herbarium (CHO) |
| Gyeongsangbuk-do, Gyeongju-si, Naenam-myeon | GJ | CHGJ2511 | Chosun University Herbarium (CHO) |
| Gangwon-do, Goseong-gun, Toseong-myeon | GO | CHGO2533 | Chosun University Herbarium (CHO) |
| Jeollanam-do, Gurye-gun, Masan-myeon | GR | CHGR2518 | Chosun University Herbarium (CHO) |
| Gyeongsangnam-do, Jinju-si | JJ | CHJJ2509 | Chosun University Herbarium (CHO) |
| Gyeonggi-do, Pocheon-si, Soheul-eup | PC | CHPC2530 | Chosun University Herbarium (CHO) |
| Seoul, Nowon-gu, Junggye-dong | SE | CHSE2510 | Chosun University Herbarium (CHO) |
| Gyeongsangbuk-do, Sangju-si, Yeonwon-dong | SG | CHSG2511 | Chosun University Herbarium (CHO) |
| Gyeongsangbuk-do, Seongju-gun, Gacheon-myeon | SJ | CHSJ2507 | Chosun University Herbarium (CHO) |
| Chungcheongnam-do, Seosan-si, Seongyeon-myeon | SS | CHSS2515 | Chosun University Herbarium (CHO) |
| Ulsan, Buk-gu | US | CHUS2510 | Chosun University Herbarium (CHO) |
| Gangwon-do, Wonju-si, Sicheong-ro | WJ | CHWJ2518 | Chosun University Herbarium (CHO) |
| Gyeongsangbuk-do, Yeongdeok-gun, Yeongdeok-eup | YD | CHYD2509 | Chosun University Herbarium (CHO) |
| Gangwon-do, Yanggu-gun, Bangsan-myeon | YG | CHYG2505 | Chosun University Herbarium (CHO) |

Table S2. Pairwise genetic differentiation (FST; lower diagonal) and associated P-values from permutation tests (upper diagonal).

|  | AH | BY | CC | CY | DE | DJ | ES | GJ | GO | GR | JJ | PC | SE | SG | SJ | SS | US | WJ | YD | YG |
| --- | --- | --- | --- | --- | --- | --- | --- | --- | --- | --- | --- | --- | --- | --- | --- | --- | --- | --- | --- | --- |
| AH | * | 0.000 | 0.000 | 0.000 | 0.000 | 0.000 | 0.000 | 0.000 | 0.000 | 0.000 | 0.000 | 0.000 | 0.000 | 0.000 | 0.000 | 0.000 | 0.000 | 0.000 | 0.000 | 0.00901 |
| BY | 0.044 | * | 0.000 | 0.000 | 0.000 | 0.000 | 0.000 | 0.000 | 0.000 | 0.000 | 0.000 | 0.000 | 0.000 | 0.000 | 0.000 | 0.000 | 0.000 | 0.000 | 0.000 | 0.000 |
| CC | 0.035 | 0.025 | * | 0.000 | 0.000 | 0.20721 | 0.4684 | 0.000 | 0.00901 | 0.000 | 0.10811 | 0.28829 | 0.00901 | 0.06306 | 0.000 | 0.000 | 0.01802 | 0.22523 | 0.06306 | 0.1982 |
| CY | 0.110 | 0.105 | 0.078 | * | 0.000 | 0.000 | 0.000 | 0.000 | 0.000 | 0.000 | 0.000 | 0.000 | 0.000 | 0.000 | 0.00901 | 0.000 | 0.02703 | 0.000 | 0.000 | 0.04505 |
| DE | 0.121 | 0.105 | 0.088 | 0.159 | * | 0.000 | 0.000 | 0.000 | 0.000 | 0.000 | 0.000 | 0.000 | 0.000 | 0.000 | 0.000 | 0.000 | 0.000 | 0.000 | 0.000 | 0.00901 |
| DJ | 0.045 | 0.038 | 0.012 | 0.078 | 0.085 | * | 0.000 | 0.000 | 0.000 | 0.000 | 0.18919 | 0.23423 | 0.01802 | 0.33333 | 0.000 | 0.000 | 0.0991 | 0.27027 | 0.000 | 0.16216 |
| ES | 0.036 | 0.029 | 0.006 | 0.077 | 0.083 | 0.020 | * | 0.000 | 0.000 | 0.000 | 0.58559 | 0.01802 | 0.000 | 0.00901 | 0.000 | 0.000 | 0.02703 | 0.2973 | 0.00901 | 0.02703 |
| GJ | 0.055 | 0.043 | 0.026 | 0.100 | 0.103 | 0.032 | 0.028 | * | 0.000 | 0.000 | 0.000 | 0.000 | 0.000 | 0.000 | 0.000 | 0.000 | 0.000 | 0.000 | 0.000 | 0.000 |
| GO | 0.095 | 0.081 | 0.043 | 0.117 | 0.114 | 0.055 | 0.051 | 0.066 | * | 0.00901 | 0.000 | 0.02703 | 0.000 | 0.000 | 0.000 | 0.000 | 0.000 | 0.00901 | 0.000 | 0.0991 |
| GR | 0.032 | 0.024 | 0.014 | 0.093 | 0.095 | 0.025 | 0.019 | 0.035 | 0.075 | * | 0.000 | 0.000 | 0.000 | 0.000 | 0.000 | 0.000 | 0.000 | 0.000 | 0.000 | 0.000 |
| JJ | 0.038 | 0.029 | 0.013 | 0.090 | 0.093 | 0.021 | 0.008 | 0.029 | 0.057 | 0.067 | * | 0.16216 | 0.02703 | 0.13514 | 0.00901 | 0.000 | 0.03604 | 0.13514 | 0.06306 | 0.02703 |
| PC | 0.040 | 0.031 | 0.007 | 0.081 | 0.078 | 0.015 | 0.013 | 0.032 | 0.032 | 0.034 | 0.015 | * | 0.00901 | 0.0991 | 0.000 | 0.000 | 0.000 | 0.88288 | 0.08108 | 0.88288 |
| SE | 0.064 | 0.061 | 0.038 | 0.111 | 0.111 | 0.044 | 0.039 | 0.062 | 0.073 | 0.084 | 0.044 | 0.038 | * | 0.01802 | 0.000 | 0.000 | 0.03604 | 0.00901 | 0.000 | 0.09009 |
| SG | 0.036 | 0.035 | 0.011 | 0.080 | 0.078 | 0.011 | 0.012 | 0.033 | 0.046 | 0.060 | 0.014 | 0.010 | 0.041 | * | 0.000 | 0.000 | 0.000 | 0.06306 | 0.02703 | 0.08108 |
| SJ | 0.069 | 0.056 | 0.040 | 0.111 | 0.117 | 0.044 | 0.040 | 0.060 | 0.078 | 0.102 | 0.038 | 0.041 | 0.073 | 0.041 | * | 0.000 | 0.00901 | 0.000 | 0.000 | 0.00901 |
| SS | 0.046 | 0.039 | 0.018 | 0.092 | 0.096 | 0.031 | 0.023 | 0.039 | 0.060 | 0.068 | 0.022 | 0.024 | 0.053 | 0.028 | 0.048 | * | 0.000 | 0.000 | 0.000 | 0.000 |
| US | 0.042 | 0.034 | 0.014 | 0.091 | 0.096 | 0.027 | 0.017 | 0.045 | 0.073 | 0.088 | 0.019 | 0.021 | 0.048 | 0.025 | 0.055 | 0.032 | * | 0.000 | 0.000 | 0.00901 |
| WJ | 0.036 | 0.036 | 0.007 | 0.078 | 0.085 | 0.012 | 0.008 | 0.032 | 0.044 | 0.076 | 0.011 | 0.002 | 0.040 | 0.010 | 0.045 | 0.019 | 0.021 | * | 0.000 | 0.05405 |
| YD | 0.056 | 0.047 | 0.021 | 0.099 | 0.100 | 0.029 | 0.021 | 0.047 | 0.050 | 0.076 | 0.031 | 0.021 | 0.058 | 0.025 | 0.058 | 0.035 | 0.040 | 0.059 | * | 0.15315 |
| YG | 0.047 | 0.038 | 0.009 | 0.092 | 0.087 | 0.022 | 0.017 | 0.034 | 0.033 | 0.052 | 0.025 | 0.001 | 0.044 | 0.011 | 0.042 | 0.031 | 0.026 | 0.033 | 0.027 | * |


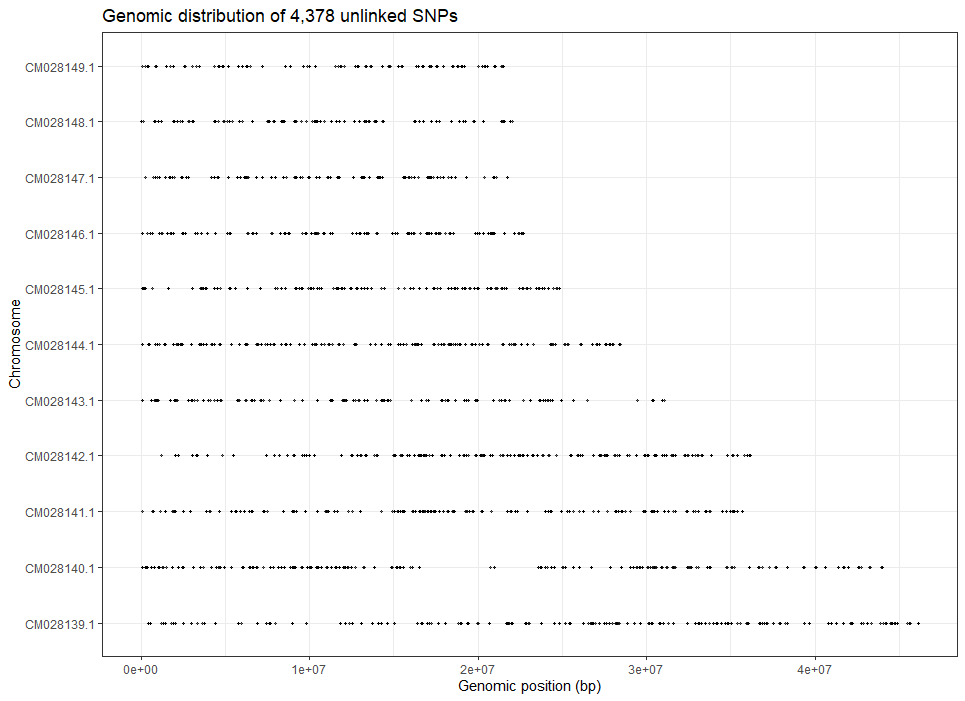


Figure S1. Genomic distribution of 4,378 unlinked SNPs across chromosomes of *Corylus heterophylla*. SNPs are widely dispersed across the genome, reflecting the selection of a single SNP per RAD locus to minimize linkage.


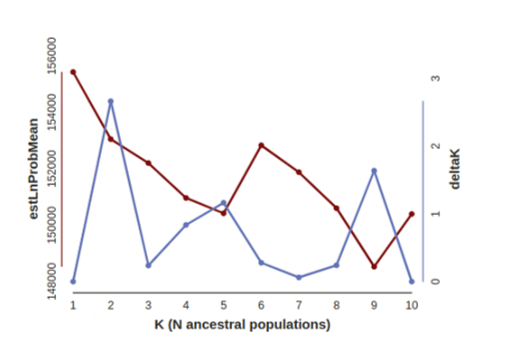


Figure S2. Summary of Structure Harvester results. The red line indicates the log probability of the data [LnP(K)] for each K, whereas the blue line represents ΔK values. The most likely number of clusters is inferred from the peak in ΔK.

a)


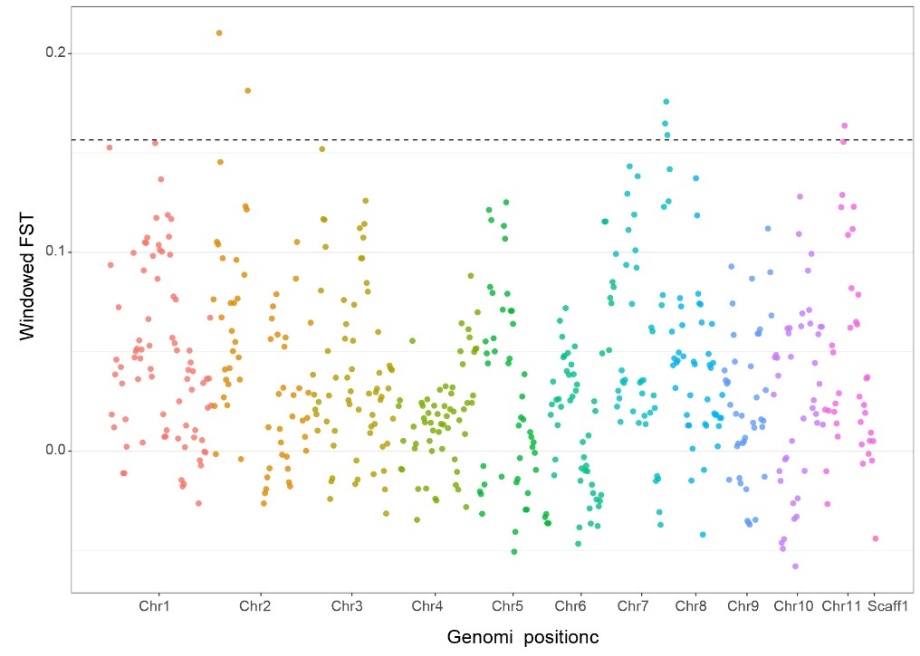


b)


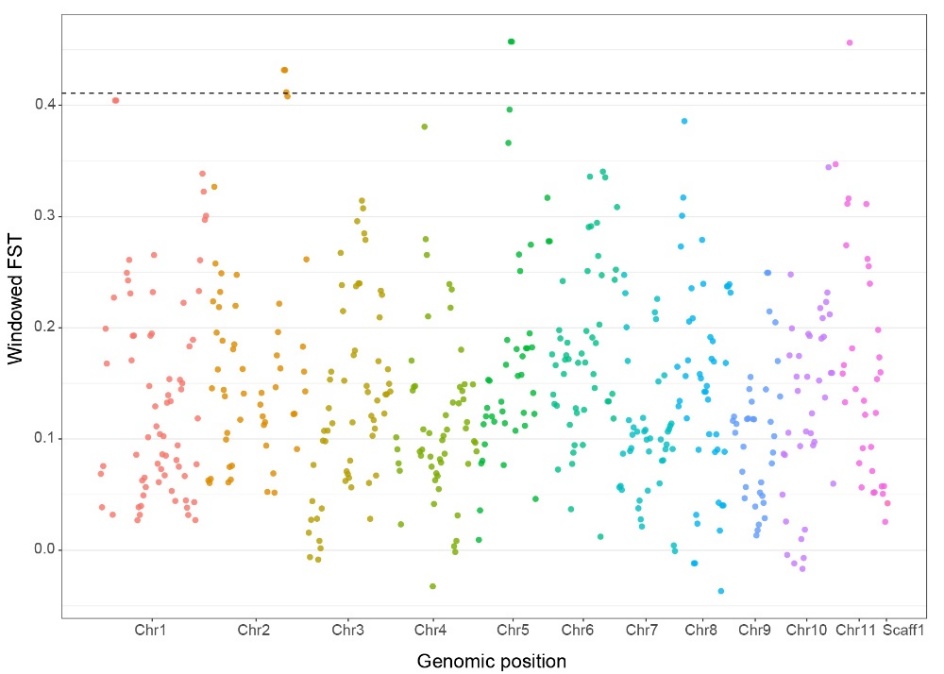


Figure S3. Genome-wide distribution of genetic differentiation (FST) in *Corylus heterophylla*. Manhattan plots illustrate pairwise FST for (a) DE vs. CY, representing the maximum observed genome-wide mean FST, and (b) US vs. SE, representing a mean FST closest to the global average across all comparisons. Values were calculated using a 2-Mb sliding window with a 0.5-Mb step. Each point denotes the mean FST of a window, with colors alternating by chromosome (Chr1–Chr11 and scaffolds). The dashed horizontal line represents the upper 5% (or 1%, as applicable) quantile threshold, highlighting genomic regions with elevated differentiation.

a)


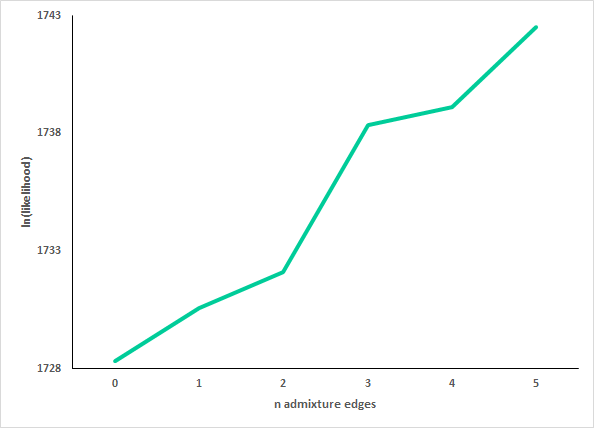


b)


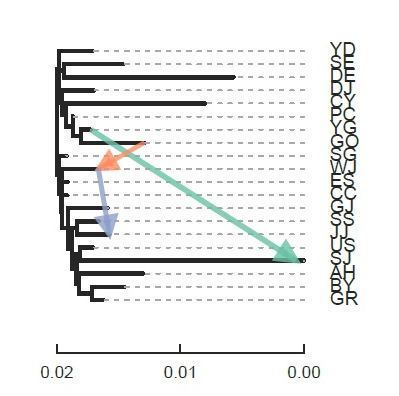


Figure S4. TreeMix analysis of 20 populations of *Corylus heterophylla*. a) Log-likelihood values for models with increasing numbers of migration edges (m = 0-5), showing incremental improvement in model fit with additional migration events. b) Maximum likelihood tree inferred from genome-wide SNP data. Migration edges were evaluated but no clear or consistent migration events were detected. Branch lengths are proportional to genetic drift.
